# A Rapid Grading Method for Beef Appearance Quality Based on Smartphone Imaging and ImageJ

**DOI:** 10.3390/foods15040709

**Published:** 2026-02-13

**Authors:** Peng Hu, Pengfei Du, Yanxia Xing, Yiyi Li, Weimin Ma, Weizhen Xu, Weiting Wang

**Affiliations:** 1 Institute of Agro-Product Processing and Nutrition, Shandong Academy of Agricultural Sciences, Jinan 250100, China; hp19812005@163.com (P.H.); dupengfei2011@163.com (P.D.); 2 College of Food Science and Engineering, Shandong Agricultural Engineering College, Zibo 255300, China; z2014007@sdaeu.edu.cn; 3 School of Food and Liquor Engineering (School of Wuliangye Baijiu), Sichuan University of Science and Engineering, Yibin 644000, China; liyy_d@163.com; 4 Shandong Aohe Biotechnology Co., Ltd., Liaocheng 252600, China; 15562179270@163.com

**Keywords:** beef rib eye muscle, beef grading, marbling, ImageJ software, non-destructive evaluation, digital grading

## Abstract

The grading of beef appearance quality is crucial for standardizing market circulation and promoting the upgrading of the beef cattle industry. China’s current beef quality grading system, which relies primarily on human sensory-based visual assessment with marbling and meat color as core parameters, suffers from strong subjectivity, low efficiency, and large errors. This study proposes a rapid grading method for beef rib eye muscle using smartphone imaging combined with ImageJ software. Standardized images were acquired, and ImageJ was employed for grayscale conversion, threshold segmentation, and morphological processing to extract length, width, area, and marbling proportion. The R, G, B color channels were separated to calculate the R/(R + G + B) color ratio. Pearson correlation analysis showed that the ImageJ results were highly consistent with manual measurements (correlation coefficients > 0.97), indicating good reliability. A five-level grading standard (A1–A5) was established, characterized by low cost, simple operation, and objective results. It provides an economical technical solution for beef quality grading and facilitates the intelligent development of the industry. It should be noted that this experimental grading model has only been validated under the specific experimental conditions of this study, and further verification is required for broader application.

## 1. Introduction

As a globally important source of high-quality protein, the appearance quality of beef is directly related to consumer purchasing intentions and the realization of market value, and it occupies a crucial position in the food industry [1,2,3]. Establishing a scientific beef grading system is a key measure to drive market consumption upgrading, standardize beef circulation practices, and advance the high-quality development of the beef cattle industry. The grading basis of China’s current national standard GB/T 29392-2022 Meat Quality Grading of Livestock and Poultry-Beef [4] mainly includes marbling, physiological maturity, muscle color, and fat color. Among these, the most important indicator is the abundance of marbling [5]. These indicators are not only the core basis for determining meat quality grades but also have a significant correlation with beef tenderness, flavor, and nutritional value [6].

However, current global beef grading still relies on human sensory-based visual assessment, which suffers from inherent drawbacks: strong operator subjectivity, low on-site grading efficiency, and poor consistency in standard implementation [7]. These shortcomings result in unstable grading outcomes and fail to meet the automated and intelligent production requirements of the modern beef industry.

In recent years, scholars have explored non-destructive beef grading technologies. These include machine vision and near-infrared spectroscopy [8,9,10,11,12,13,14,15,16]. However, these technologies have obvious limitations. Conventional machine vision relies heavily on manual feature extraction. It is also constrained by complex sample environments. Near-infrared spectroscopy and hyperspectral imaging have their own drawbacks. They require bulky professional equipment. The cost of this equipment is high. This leads to high testing costs. It also results in poor on-site adaptability. These factors have a negative impact. They limit the large-scale promotion of these technologies. The promotion is mainly restricted to slaughterhouses. It is also limited to small- and medium-sized production enterprises.

With the iterative upgrading of computer vision technology and the widespread adoption of high-resolution camera functions in smartphones, mobile terminal-based image acquisition and analysis technology has demonstrated significant application potential in the agricultural and food sectors [17,18,19]. Mobile phone photography, with advantages such as strong portability, low cost, and simple operation, can quickly obtain high-resolution image data, providing an efficient data collection solution for non-contact and non-destructive testing of food quality [20,21,22]. As an open-source image processing software with rich analysis tools, ImageJ has been applied to the prediction of intramuscular fat content in beef [23]. However, the integrated application of smartphone image acquisition and ImageJ quantitative analysis in beef appearance quality grading remains largely unexplored, and a complete, standardized grading system tailored for industrial field application has not yet been established.

According to the industry standard NY/T 676-2003 Beef Grading Standards [24], the rib eye muscle cut of beef is classified into four grades—Grade Special, Grade 1, Grade 2 and Grade 3—based on its color and texture characteristics. Among them, Grade Special rib eye muscle features bright red muscle with white or milky-white fat of distinct luster, and its marbling is abundant and evenly distributed. Grade 1 rib eye muscle features red muscle, white or slightly yellow fat, and relatively abundant marbling. Grade 2 rib eye muscle exhibits pale red muscle, light yellow fat, and somewhat scattered marbling. Grade 3 rib eye muscle is characterized by dark red muscle, yellow fat, and sparse, unevenly distributed marbling. These grading standards serve as an important basis for evaluating beef quality but rely on subjective visual inspection prone to errors.

Against the above research gaps and industrial demands, this study takes beef rib eye muscle as the research object. The core research objective is to address the critical limitations of traditional manual grading and existing high-cost detection technologies by developing a novel, objective, low-cost and field-adaptable beef grading system through smartphone-based high-resolution image acquisition combined with open-source ImageJ quantitative analysis. This work aims to establish a standardized grading criterion for beef classification into five grades (A1–A5) and realize the quantitative evaluation of key appearance quality indicators including marbling, muscle color and tissue dimensions. This study’s novelty lies in the following: (1) consumer-grade smartphones instead of expensive devices; (2) simple ImageJ tools over complex models; and (3) integrating morphology, marbling, and color ratio for comprehensive grading. The proposed method is expected to provide technical support for meat processing enterprises, slaughterhouses, regulatory authorities and small- and medium-scale beef producers, facilitating the standardization and intelligent transformation of the beef grading process. This study offers a low-cost and easy-to-operate solution for the rapid detection of beef quality, which is expected to facilitate the upgrading of the beef industry toward standardization and intelligence. It also has significant theoretical value and practical importance for improving the beef grading system and promoting the high-quality development of the industry.

## 2. Materials and Methods

### 2.1. Materials and Instruments

The experiment used cross-sectional samples of the rib eye muscle. The rib eye muscle was from the longissimus dorsi. It was taken between the 6th and 7th ribs. These samples were purchased from Shandong Aohe Biotechnology Co., Ltd. (Liaocheng, China). A total of 50 independent longissimus dorsi samples were collected. They came from 30 healthy black-haired beef cattle. Among these cattle, there were 15 heifers and 15 steers. Their ages ranged from 18 to 24 months. The cattle were fed with three typical commercial diets. The first diet was com-soybean meal-based. The second was a forage–concentrate mix. The third was a high-grain diet. These diets were used to cover natural variations in marbling and muscle development. Each cattle contributed 1 to 2 samples. One sample was taken from the left 6th–7th rib position, or one was taken from the right 6th–7th rib position. This was done to ensure the representativeness of the samples.

**Figure 1:**
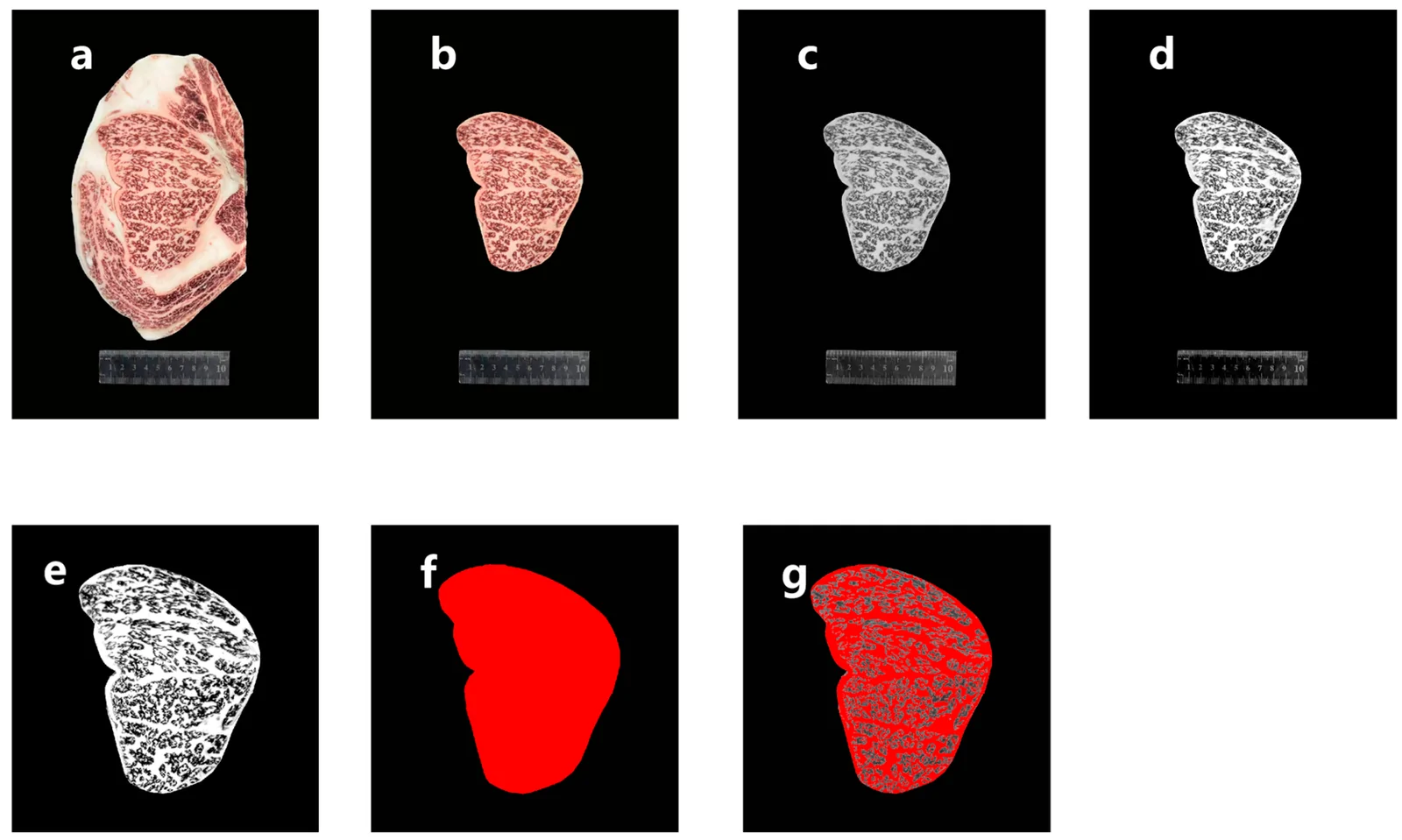
Image processing of beef rib eye muscle: (**a**) original image of beef rib segment (containing multiple fats); (**b**) isolated rib eye muscle (cropped to remove redundant fats); (**c**) grayscale converted image; (**d**) contrast-adjusted grayscale image; (**e**) cropped rib eye muscle; (**f**) threshold-segmented image of rib eye muscle (entire muscle in red); (**g**) threshold-segmented image of marbling (marbling in red).

The samples were vacuum packaged when received. After unpackaging, they were stored at 4 °C and processed within 2 h. The size specifications of the samples were standardized as follows: cross-sectional length 8–15 cm, width 5–10 cm, and thickness controlled at 1.0 ± 0.1 cm (trimmed with a surgical blade to avoid imaging errors caused by uneven thickness). Note that Figure 1a shows the original beef rib segment containing multiple adjacent muscles, but only the isolated rib eye muscle was selected for subsequent imaging and analysis (Figure 1b–g).

Sample selection criteria included the following: (1) freshness—collected within 24 h post-slaughter, free of discoloration, deterioration, or surface moisture loss; (2) uniformity—intact rib eye cross-sections without fascia tearing or excess adipose tissue, ensuring consistent morphology; and (3) representativeness—covering a wide range of marbling (from <10% to >70%) abundance and muscle color to ensure the grading model’s applicability.

A smartphone (Apple iPhone 16 Pro, 48 MP main camera, fixed focal length, default automatic exposure and white balance, Apple Inc., Cupertino, CA, USA), a computer with ImageJ software (Version 1.54d, National Institutes of Health, Bethesda, MD, USA), and a mobile phone fixed support (Tripod Pro, Shenzhen, China) were used.

### 2.2. Experimental Methods

#### 2.2.1. Image Acquisition

Fifty rib eye muscle samples were randomly selected for image acquisition (20 samples for grading model establishment and 30 samples for model validation). The specific steps are as follows:

Trim the sample thickness to 1.0 ± 0.1 cm. Then, place the sample on a matte black experimental bench. Position a reference scale under the sample. Fix the smartphone on a professional mobile phone support. The shooting distance is 30 cm from the sample surface. The shooting angle is vertical. It is 90° relative to the sample cross-section.

Even lighting is essential to reduce experimental errors. Use two LED cold light sources. Each source is 5 W, with a wavelength of 5500 K and a powder of 10 W. Place the two light sources symmetrically. They are 20 cm away from both sides of the sample. The illumination intensity is 800 lux.

Adjust the light sources. Make them parallel to the sample surface. This eliminates reflections and shadows. It further reduces experimental errors.

Capture the sample images. Save them in JPEG format. The resolution is 4032 × 3024 pixels. This balance ensures both image quality and appropriate file size.

Control the time between sample slicing and image acquisition. It must be within 10–15 min. This avoids color changes. Color changes are caused by oxidation.

The photograph results are shown in Figure 1. The steps to obtain the parameters related to the rib eye muscle are as follows:

1. Launch ImageJ software and import the captured image, then separate the rib eye muscle from the rest of the image (Figure 1b).
2. Used the straight-line tool to draw a line of a specific length (e.g., 5 cm) along the scale of the vernier caliper in the image. Calibrate the scale through “Analyze” → “Set Scale” (known distance: 5, unit of length: cm, and global), to convert pixel values into actual distances.
3. Adjust the image contrast to distinguish the rib eye muscle from the black background and enhance the marbling contrast. Convert the image to grayscale through “Image” → “Type” → “8-bit”, then automatically adjust the contrast via “Image” → “Adjust” → “Brightness/Contrast” → “Auto” (Figure 1c,d).
4. Crop the image to retain only the portion of the rib eye muscle, which facilitates area calculation and enhances the esthetics of image analysis. Use the rectangular selection tool to select the rib eye muscle, then crop it via “Image” → “Crop” (Figure 1e).
5. Adjust the image threshold. Modify the threshold through “Image” → “Adjust” → “Threshold” to ensure the entire rib eye muscle appears red. Repeat this step to adjust the threshold further, making the marbling also appear red (Figure 1f,g).
6. Set the required measurement parameters (such as area, etc.) via “Analyze”→ “Set Measurements”, then use “Analyze” → “Analyze Particles” to measure the required parameters. The measured particle size range was set to 0.5~infinity, where 0.5 was determined based on pre-experiments: it is the minimum size of effective marbling particles, which can filter out tiny impurities (e.g., dust and fascia fragments) and background noise, ensuring the accuracy of marbling proportion calculation.

#### 2.2.2. Color Characteristics of Rib Eye Muscle

The steps for obtaining the color parameters of rib eye muscle are as follows:

1. Launch ImageJ software, import the captured images, and separate rib eye muscle from the background.
2. Decompose the color image of rib eye muscle into red (R), green (G) and blue (B) channels via the path “Image” → ”Color” → ”Split Channels”.
3. Extract the R, G and B intensity values of rib eye muscle through “Analyze” → ”Tools” → ”Color Histogram”.
4. Import the data into Excel to calculate the R/(R + B + G) ratio.

RGB was selected based on three core justifications: (1) compatibility—as smartphones’ native output, it enables direct color extraction without complex conversion, ensuring simplicity and minimal data loss; (2) practicality—its R/(R + G + B) ratio effectively quantifies red intensity variations (dark red → pale red → bright red) aligned with beef grading criteria; and (3) accessibility—ImageJ natively supports RGB channel separation, making the method low-cost and reproducible. However, RGB has limitations compared to CIELAB: (1) device/lighting sensitivity—RGB values vary with camera sensors, color temperature, and illuminance, causing inconsistencies; (2) perceptual non-uniformity—unlike CIELAB’s ΔE* (reflecting human visual perception), RGB lacks a linear correlation with subjective color differences; and (3) illuminant dependence—CIELAB’s L*, a*, b* values are device/illuminant-independent, while RGB shifts under variable lighting. These were mitigated by strictly controlling imaging conditions (fixed color temperature, illuminance, and camera settings; Section 2.2.1), ensuring R, G, B reliability within the standardized framework.

#### 2.2.3. Manual Measurement of the Length, Width and Area of Rib Eye Muscle

1. Sample Pretreatment

The beef rib eye muscle was fully separated, and the fascia and adipose tissue attached to its surface were carefully trimmed off using a surgical blade to ensure the original morphology of the muscle tissue remained intact. The treated samples were then placed flat on a black non-reflective laboratory bench and allowed to stand for 10 to 15 min to eliminate tissue deformation stress without altering muscle appearance—consistent with the sample conditions in Section 2.2.1.

2. Length and Width Measurement

A calibrated vernier caliper (accuracy 0.02 mm, same as the reference standard for image-based measurements) was used.

Length: A vernier caliper with an accuracy of 0.02 mm, calibrated by a metrological institution, was selected. The maximum length was determined using the three-point comparison method, which involves measuring the longest part and positions 1 cm away from both sides of it along the main direction of the muscle fibers of the rib eye muscle. During the measurement, the external measuring jaws of the vernier caliper were firmly attached to the muscle tissue and positioned perpendicular to the measuring surface. The measurement was repeated three times with a 5 min interval between each measurement, and the maximum value was taken as the length L (unit: cm).

Width: On the cross-section perpendicular to the direction of muscle fibers, the three-point comparison method was used in the same way. The widest part was selected for measurement, and the width was recorded as W (unit: cm). Before each measurement, the measuring surface of the caliper must be wiped with absolute ethanol to eliminate grease interference.

#### 2.2.4. Beef Grading Criteria Establishment

Grading Basis: Refer to industry standard NY/T 676-2003 Beef Grading Standards. Integrate three core parameters. The parameters are rib eye area, marbling percentage, and R/(R + G + B) ratio. They are measured by smartphone–ImageJ.

Sensory Evaluation Validation: Three senior meat quality assessors participated. Each had ≥8 years of experience. They were trained per NY/T 676-2003. They conducted blind sensory grading. The grading covered marbling muscle color. There were 2 replicates. Discrepancies were resolved via group discussion. The coefficient of variation (CV) was <5%.

Grade Definition: Divided into five levels (A1–A5). The division is based on Gaussian fitting. The fitting used 20 training samples (4 samples per grade). It also referred to sensory validation results. Refine thresholds. Maximize intergrade discrimination.

Validation: Use 30 independent samples. There were 6 samples per grade. Verify the model’s accuracy.

3. Area Measurement

The grid paper method was used to determine the area of irregular samples. The grid paper (side length of single grid a = 0.5 cm, area a^2^ = 0.25 cm^2^) was laid flat on a black laboratory bench, and the rib eye muscle was gently placed on the paper surface to ensure it was securely attached. The outer contour of the rib eye muscle was then slowly traced with a pencil. The number of grids completely covered within the contour was counted and recorded as N1. Grids with more than half of their area covered by the contour line were recorded as N2, while those with less than half coverage were ignored. Each grid had a side length of a (unit: cm), and the area of a single grid was a^2^. Therefore, the total area of the rib eye muscle was calculated using the following formula:

S = (N1 + N2) × a^2^ (1)

For each group of samples, five parallel measurements were conducted, and the arithmetic mean value along with the standard deviation were calculated. Median values were also computed for verification, and the results were consistent with the mean, confirming robustness.

### 2.3. Data Processing

After manually measuring the length, width and area of the rib eye muscle, Gaussian distribution fitting was used to eliminate random outliers (not normalize data), ensuring the retention of real measured values. The justification for adopting Gaussian fitting is as follows: (1) morphological parameters (length, width, and area) and marbling proportion of beef rib eye muscle, as biological traits, typically follow a continuous normal distribution in large sample populations, which aligns with the theoretical premise of Gaussian fitting; and (2) Gaussian fitting can effectively mitigate the impact of random outliers (e.g., measurement errors caused by manual operation or equipment noise) without discarding valid data, ensuring the robustness of results. For the measurement combining mobile phone photography with ImageJ software, the collected beef rib eye muscle images were preprocessed with ImageJ software according to the methods described in Section 2.2.1 and Section 2.2.2. The processing steps included setting the image scale, adjusting image contrast and modifying the image threshold. The Analyze Particles plugin was used to obtain the length, width, area and marbling ratio of the rib eye muscle. The data were imported into Origin for Gaussian distribution fitting analysis, followed by Pearson analysis to determine the correlation between different measurement methods. No data transformation was performed for the Pearson test, as the data met the normal distribution requirement verified by Gaussian fitting. The samples were tested independently (*n* = 3).

## 3. Results and Discussion

### 3.1. Comparison of Rib Eye Muscle Length, Width, and Area Measured Manually Versus Using ImageJ Software

The manual measurement of the length, width and area of beef rib eye muscle was conducted in accordance with the method described in Section 2.2.3. Fifty beef rib eye muscle samples were randomly selected. Surface fascia and adipose tissue were carefully removed with a surgical blade to preserve the muscle’s original morphology. The isolated samples were placed flat on a black non-reflective bench and allowed to stand to relieve tissue deformation stress. Subsequently, a vernier caliper with an accuracy of 0.02 mm was used to measure the length and width, resulting in an average length of (11.997 ± 0.082) cm and an average width of (7.709 ± 0.240) cm for the rib eye muscle. The area of the rib eye muscle was determined using the grid paper method: the processed samples were smoothly affixed to the grid paper, and their outer contours were precisely traced with a pencil. The grid paper had a minimum unit side length a = 0.5 cm, with a single grid area of 0.25 cm^2^. For statistical purposes, fully covered grids were recorded as N1, grids with more than half their area covered by the contour were recorded as N2, and grids with less than half coverage were discarded. According to the calculation in formula (1), the average rib eye muscle area was calculated to be (76.522 ± 2.556) cm^2^.

**Figure 2:**
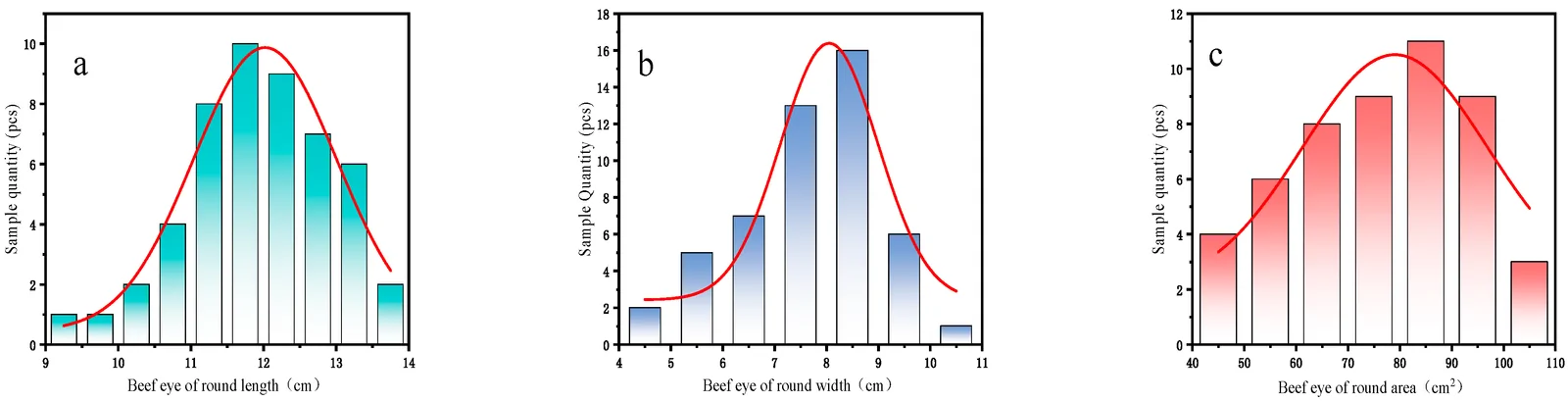
Gaussian distribution plots of length, width, and area of beef rib eye muscle samples (data from image processing technology, no normalization performed); (**a**) Gaussian distribution of sample length; (**b**) Gaussian distribution of sample width; (**c**) Gaussian distribution of sample area.

The apparent length, width and area of rib eye muscle were measured using mobile phone photography combined with image processing technology based on ImageJ software, following the method described in Section 2.2.1. Samples for image acquisition were processed identically to manual measurement (fascia trimmed and 10–15 min standing) to ensure comparability. The samples were photographed with a mobile phone at a fixed shooting distance and under consistent lighting conditions to ensure that the images were clear and complete. The captured images were imported into ImageJ software, and the Analyze Particles function was used to obtain the apparent morphological parameters of the rib eye muscle, such as the length, width, and area. To enhance the reliability and accuracy of the data, Gaussian fitting was employed for the statistical analysis of the image processing results. The Gaussian distribution can effectively identify and remove abnormal data resulting from individual characteristic differences, thereby obtaining a data interval closer to the true values and accurately reflecting the common rules of the dimensional characteristics of most rib eye muscles. The dimensional parameters of the rib eye muscle obtained by image processing technology were: length of (12.019 ± 0.062) cm, width of (8.050 ± 0.153) cm, and area of (79.065 ± 3.006) cm^2^. The specific measurement results are shown in Figure 2.

### 3.2. Correlation Analysis of Rib Eye Muscle Length, Width and Area of Beef Measured by Different Methods

According to the measurement results in Section 3.1, the average length, width and area of the beef rib eye muscle measured by the vernier caliper with an accuracy of 0.02 mm were (11.997 ± 0.082) cm, (7.709 ± 0.240) cm and (76.522 ± 2.556) cm^2^, respectively. The length, width and area of the beef rib eye muscle obtained by the image processing technology were (12.019 ± 0.062) cm, (8.050 ± 0.153) cm and (79.065 ± 3.006) cm^2^, respectively. There were certain differences between the results of manual measurement and those obtained by combing mobile phone photography with ImageJ software analysis. The reasons are as follows:

1. Manual measurement used the vernier caliper method and the grid paper method, which depended on physical contact and manual counting. The force applied during caliper measurement and the subjective judgment involved in grid paper counting both introduce errors.
2. Image processing technology was based on image pixel recognition and algorithmic calculation, and it was greatly influenced by factors such as the sample placement angle, shooting light and shadow conditions, and software parameter settings. The different principles of the two methods resulted in data deviations.
3. Data processing in manual measurement mostly involved simple calculations of mean and standard deviation, with limited ability to identify outliers. In contrast, image processing results were statistically analyzed by Gaussian fitting, which could effectively eliminate data significantly deviating from the true range.

**Figure 3:**
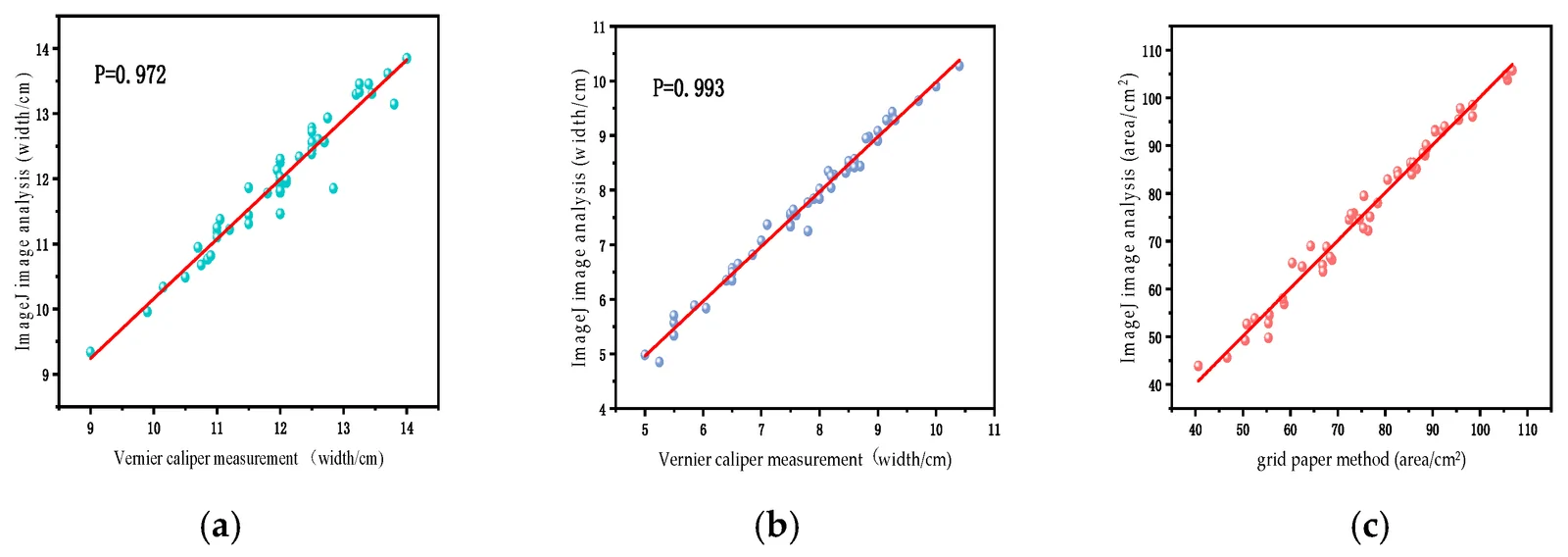
Correlation analysis of beef rib eye muscle length, width and area measured by different methods: (**a**) correlation between beef rib eye muscle length measured by ImageJ software and vernier caliper; (**b**) correlation between beef rib eye muscle width measured by ImageJ software and vernier caliper; (**c**) correlation between beef rib eye muscle area measured by ImageJ software and grid paper method. All correlations are statistically significant (*p* < 0.01).

Figure 3 shows the results of the correlation analysis between manual measurement and the statistics obtained by combining mobile phone photography with ImageJ software. Data analysis revealed a correlation coefficient of 0.972 (*p* < 0.01) between manually measured beef rib eye muscle length with a vernier caliper and the length calculated via mobile phone photography combined with ImageJ software. The scatter plot shows a uniform distribution, indicating an extremely significant correlation between the two methods. For the beef rib eye muscle width, the correlation coefficient between manual vernier caliper measurements and mobile phone photography–ImageJ software statistics was as high as 0.993 (*p* < 0.01), with data points tightly clustered around the fitted line, indicating a high degree of consistency. Regarding the beef rib eye muscle area, the correlation coefficient between the manual grid paper measurement and the values obtained from the mobile phone photography–ImageJ software combination was 0.990 (*p* < 0.01). Although the overall correlation was extremely strong, slight data dispersion was observed across the large-area range, which is presumed to result from the cumulative effect of measurement variations in length and width. All correlation coefficients of the measured indicators exceeded 0.97, confirming that the image processing technology combining mobile phone photography with ImageJ software can serve as a highly efficient alternative tool for measuring beef rib eye muscle dimensions, and its measurement results are highly consistent with those of traditional manual measurement. In composition, image processing technology has distinct advantages of fast measurement speed and strong objectivity. It can effectively avoid subjective errors caused by manual operations and thus holds important application value. Further statistical analysis of the data using Gaussian distribution fitting can more accurately eliminate outliers, obtain the true characteristic parameters of beef rib eye muscle dimensions, and enhance the reliability and accuracy of the measurement results.

### 3.3. Analysis of Beef Rib Eye Muscle Area and Marbling Percentage

**Figure 4:**
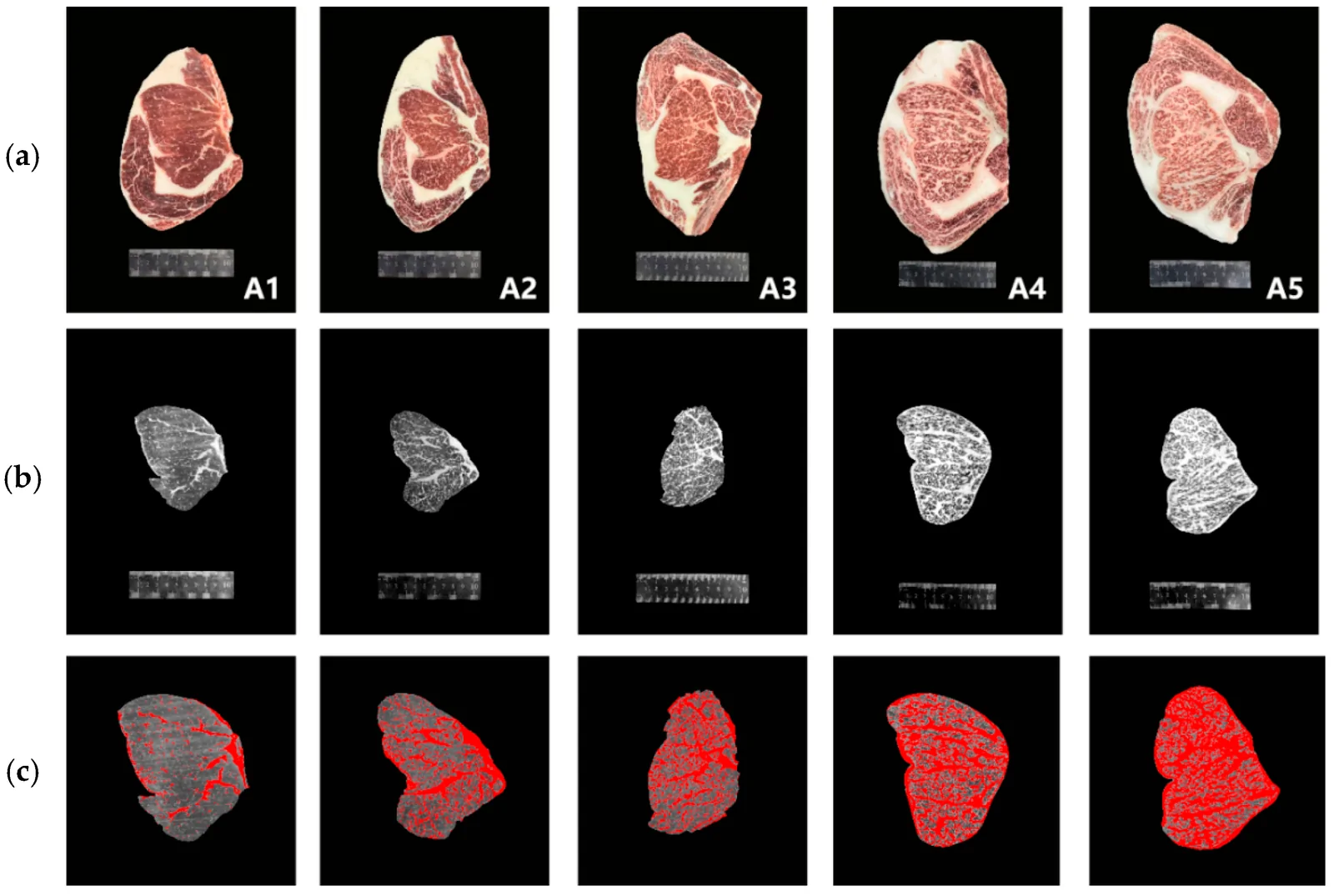
Five examples of pictures of beef rib eye muscle area and marbling: (**a**) original color image; (**b**) beef rib eye muscle; (**c**) marbling.

In the global beef quality evaluation system, although different countries and regions adopt differentiated grading standards, marbling remains a core evaluation index. As a characteristic of the interleaved distribution of intramuscular fat and muscle tissue, complex and dense marbling is regarded as an important indicator of tender, juicy and rich-flavored beef. Based on the method described in Section 2.2.1, mobile phone photography combined with ImageJ software was used to conduct quantitative analysis of the beef rib eye muscle area and marbling percentage (image-based estimation). Figure 4 shows five examples of pictures of beef samples (A1–A5, six samples per grade in total), including original color images, grayscale processed images (used to determine the rib eye muscle area) and threshold segmentation images (used to calculate marbling percentage).

The experimental results showed that Grade A1 samples had the smallest rib eye muscle area (40–50 cm^2^), with a marbling percentage of less than 10%, reflecting a low intramuscular fat content. The rib eye muscle areas of Grade A2 and Grade A3 samples increased sequentially to 50–60 cm^2^ and 60–75 cm^2^, with their marbling percentages reaching 10–30% and 30–50%, respectively, indicating a stepped increase in fat deposition. Grade A4 samples had a rib eye muscle area of 75–90 cm^2^ and a marbling percentage of 50–70%, with a significant enhancement in the distribution of fat texture. Grade A5 samples had a rib eye muscle area exceeding 90 cm^2^ and a marbling percentage of over 70%, presenting a highly developed fat network structure.

To address the representativeness of samples regarding commercial variability, the following strategies were integrated into sample collection: First, samples were sourced from Shandong Aohe Biotechnology Co., Ltd., a large-scale commercial supplier distributing across northern China, covering mainstream consumer markets (Jinan, Zibo, Liaocheng, etc.). Second, the samples were black-haired cattle, consistent with dominant production practices. Third, the marbling percentage (<10% to >70%) and rib eye muscle area (40–90 cm^2^) span the full spectrum of commercial grades—from retail-oriented low-grade to premium catering/export-grade—matching real commercial circulation variability. Fourth, samples were collected over three consecutive months to mitigate seasonal bias, as intramuscular fat content and muscle size vary slightly with commercial feeding cycles. Collectively, these measures ensured that the samples adequately represented commercial variability in production sources, breed composition, quality grades, and seasonal distribution for beef rib eye muscle.

The quantitative results of rib eye muscle area, marbling percentage and color parameters obtained through image processing technology were highly consistent with the existing beef grading standard NY/T 676-2003 (detailed in the Introduction). This consistency was verified by sensory evaluation (Section 2.2.4): A1–A2 samples matched Grade 3–Grade 2 of NY/T 676-2003, A3 matched Grade 1, and A4–A5 matched Grade Special. The progressive variation from Grade A1 to A5 clearly revealed the gradient differences in beef quality and provided a quantitative basis for constructing an objective and standardized beef grading system. A colorimeter was not used because the study focused on developing a low-cost, portable field method; colorimeters increase equipment cost and reduce on-site adaptability, which conflicts with the research objective.

### 3.4. Color Characteristics of Beef Rib Eye Muscle

To accurately measure the appearance and color characteristics of beef rib eye muscle, the samples are subjected to R, G, B channel separation following the method described in Section 2.2.2. The R, G and B intensity values of each sample are extracted, and the R/(R + G + B) ratio is calculated to serve as the color grading criterion.

**Figure 5:**
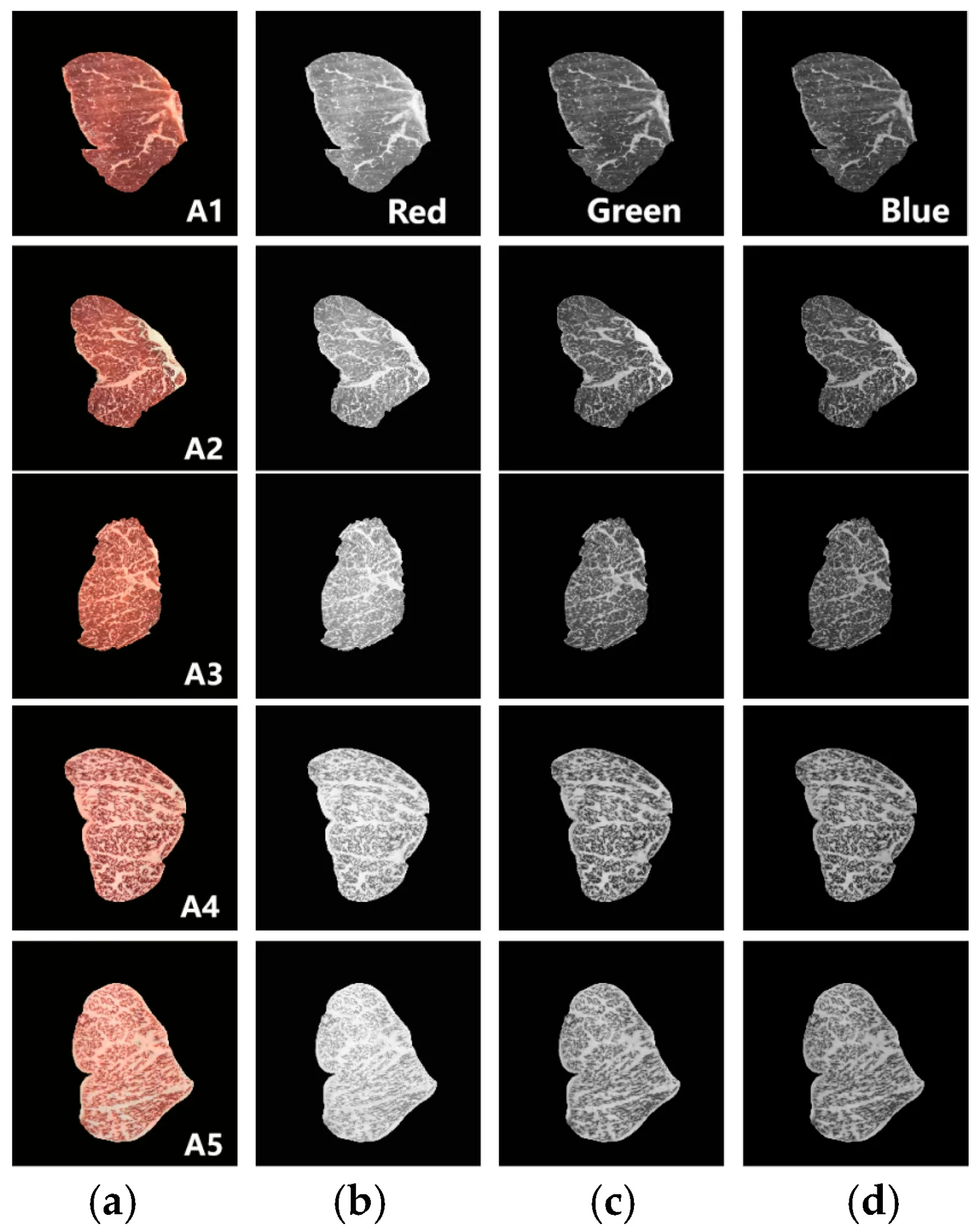
Five examples of pictures of the color characteristics of A1–A5 beef rib eye muscle: (**a**) original color image; (**b**) red channel; (**c**) green channel; (**d**) blue channel.

The experimental results are shown in Figure 5, which displays five examples of pictures of beef rib eye muscle samples (A1 to A5, one representative sample per grade) and their R, G, B channel separation effects. The “five groups” refer to the A1–A5 grading levels. Visual analysis involved observing channel pixel intensity differences and aligning with sensory evaluation results; the statistical significance of R/G/B intensity differences was confirmed by one-way ANOVA (*p* < 0.05). Visual analysis indicates that the red channel pixel values of Sample A1 are significantly higher than those of the green and blue channels, corresponding to dark red muscle with a small amount of fat texture, which matches the characteristics of Grade 3 beef rib eye muscle (dark red muscle color and sparse marbling). For Samples A2 and A3, the red channel pixel values decrease sequentially while the green and blue channel pixel values increase gradually, showing a trend of fading muscle redness and gradual emergence of fat texture. These trends are consistent with the pale red and red muscle color and corresponding texture characteristics of Grade 2 and Grade 1 beef rib eye muscle, respectively. In Samples A4 and A5, the pixel values of the green and blue channels increase significantly, enhancing the contrast of marbling and exhibiting features of dense fat texture and bright red muscle, which conform to the appearance standards of Grade Special beef rib eye muscle. Quantitative analysis of the R, G, B channel intensities of each sample shows that the calculated R/(R + G + B) radios are highly consistent with the grading standards: the ratio of Group A1 is greater than 0.49, that of Group A2 ranges from 0.49 to 0.47, Group A3 from 0.47 to 0.45, Group A4 from 0.45 to 0.43, and Group A5 is less than 0.43. The data demonstrate that from Group A1 to A5, both the R/(R + G + B) ratio and the red channel intensity exhibit a linear decreasing trend, effectively quantifying the color differences that are difficult to accurately distinguish with the naked eye. The results confirm that the R/(R + G + B) ratio analysis method, which is based on separation of the R, G, B channels, can serve as a reliable approach for the objective and quantitative evaluation of the appearance quality grading of beef rib eye muscle.

### 3.5. Beef Sample Grading Based on Smartphone Photography Combined with ImageJ Software

Based on the image processing technology integrating mobile phone photography and ImageJ software, this study establishes a beef grading system by incorporating multi-dimensional characteristics parameters of beef rib eye muscle. Using ImageJ software, morphological indicators such as the length, width and area of beef rib eye muscles were accurately extracted. By combining the R/(R + B + G) color ratio calculated through separation of the R, G and B channels, with marbling proportion data, and referring to the industry standard NY/T 676-2003, a five-level grading model (A1–A5) was established. The grading thresholds (A1–A5) were derived as follows: First, descriptive statistics and Gaussian fitting of three core measured parameters (rib eye area, marbling percentage, and R/(R + G + B) ratio) from 20 training samples (four samples per grade A1–A5) samples identified natural data stratifications corresponding to distinct quality levels. Second, these stratifications were cross-validated with manual grading results (by three senior meat quality assessors) to ensure alignment with sensory evaluation. Third, threshold values were refined iteratively to maximize the discrimination between adjacent grades, with final thresholds confirmed by minimizing classification errors on the training set. The derived thresholds were then fixed for subsequent verification on 30 independent test samples (randomly selected from the remaining 30 of the 50 total samples, with the same grade distribution as the training set).

As shown in Table 1, the grading standards were converted into machine language. Thirty beef rib eye muscle samples were randomly selected for verification. The results showed that the model’s grading accuracy reached 92.7%, its efficiency was improved by more than 80% compared to the traditional manual grading method, and errors caused by subjective judgment were eliminated, thereby achieving efficient, objective, and quantitative evaluation of beef rib eye muscle quality. Grading accuracy (92.7%) was calculated as (number of correctly classified validation samples/total validation samples) × 100% (28/30 correct classifications). Efficiency improvement (>80%) was determined by comparing the average grading time per sample: 0.7 min for proposed method vs. 4.2 min for traditional grading.

Liu, J. et al. [25] optimized the YOLOv8x model with computer vision by integrating an attention mechanism and refining the loss function, achieving 97.3% marbling consistency and 94.1% meat color grading accuracy, with an 8-fold faster detection speed than traditional methods. However, this method is highly susceptible to light interference, blood stains and fascia disrupt segmentation, tiny fat particles are indistinguishable, and it performs poorly for high intramuscular fat beef (wagyu). Hashem, M.A. et al. [26] coupled a portable near-infrared spectrometer with SHAP interpretable AI, attaining over 95% accuracy in meat color freshness grading and visualizing spectral features’ contribution to results to enhance credibility. Limitations include the need for a large number of calibration samples, inability to visualize fat distribution, and significant susceptibility to sample thickness and temperature. Song, Y.F. et al. [27] developed a polarized hyperspectral-CNN model that effectively eliminates surface reflection, yielding R^2^ = 0.94 for meat color prediction and R^2^ = 0.95 for marbling prediction, with grading accuracy significantly outperforming traditional hyperspectral approaches. Yet this method suffers from large data volume and slow processing, alongside expensive equipment (5–10 times the cost of CV devices), strict environmental temperature and humidity requirements, and complex maintenance. Dixit, Y. et al. [28] fused hyperspectral and near-infrared technologies, achieving R^2^ = 0.96 for IMF prediction and R^2^ = 0.93 for meat color a* value—an improvement of 5–8 percentage points over single technologies—and enabling comprehensive evaluation of beef’s internal and external quality. Nevertheless, the method is hampered by complex equipment, high costs, and challenging data processing. Compared with the aforementioned non-destructive beef grading methods, the method proposed in this study for marbling and meat color grading based on mobile phone imaging combined with ImageJ software features strong portability for on-site real-time grading and extremely low cost for full-scenario deployment. It can generate objective, quantitative and repeatable grading results with simple operation. It should be noted that the experimental grading model in this study has only been validated under the specific experimental conditions of the research, and further verification is required for its wider application.

## 4. Conclusions

This study successfully developed a preliminary appearance quality grading technology for beef rib eye muscle based on mobile phone photography and ImageJ software. Standardized image acquisition and software processing enabled precise extraction of morphological parameters (length, width, area, and marbling proportion) and color quantification via the R/(R + B + G) ratio, with Pearson correlation coefficients > 0.97 relative to manual measurements confirming reliability. The established five-level grading model (A1–A5) achieved 92.7% accuracy in verification, significantly improving grading efficiency and objectivity compared to traditional manual assessment. With the advantages of low cost and easy operation, this technology shows considerable promise as an intelligent and standardized grading reference for the beef industry. It is expected to promote the objective development of the beef quality grading system and holds significant practical value in enhancing industrial competitiveness and standardizing market circulation. However, this study is exploratory, with sensory/grading validation limited to specific samples and conditions; broader validation—including diverse cattle breeds, production regions, storage conditions, and alignment with more national/international standards—is therefore required to confirm generalizability. In the future, the sample size can be further expanded and combined with technologies such as near-infrared spectroscopy; in-depth research on multi-index fusion evaluation of beef quality can be conducted to further optimize and refine the grading scheme for broader applicability.

## Figures and Tables

**Table 1:** Beef grading standards based on ImageJ software image processing technology.

Grade	A1	A2	A3	A4	A5
Area of Rib Eye Muscle (cm^2^)	40~50	50~60	60~75	75~90	>90
Marbling Percentage (%)	<10%	10~30%	30~50%	50~70%	>70%
Color Ratio R/(R + G + B)	>0.49	0.49~0.47	0.47~0.45	0.45~0.43	<0.43
Number of Samples (Training/Validation)	4/6	4/6	4/6	4/6	4/6

Note: The model was established with 20 training samples and validated with 30 independent samples. Thresholds were derived from Gaussian fitting and cross-validated with sensory evaluation (NY/T 676-2003).

## Data Availability

The original contributions presented in the study are included in the article, further inquiries can be directed to the corresponding authors.
